# Comprehensive Control of Networked Control Systems with Multistep Delay

**DOI:** 10.1155/2014/814245

**Published:** 2014-07-01

**Authors:** Jie Jiang, Changlin Ma

**Affiliations:** ^1^College of Information System and Management, National University of Defense Technology, Changsha 410073, China; ^2^School of Computer, Central China Normal University, Wuhan 430079, China

## Abstract

In networked control systems with multi-step delay, long time-delay causes vacant sampling and controller design difficulty. In order to solve the above problems, comprehensive control methods are proposed in this paper. Time-delay compensation control and linear-quadratic-Guassian (LQG) optimal control are adopted and the systems switch different controllers between two different states. LQG optimal controller is used with probability 1 − *α* in normal state, which is shown to render the systems mean square exponentially stable. Time-delay compensation controller is used with probability *α* in abnormal state to compensate vacant sampling and long time-delay. In addition, a buffer window is established at the actuator of the systems to store some history control inputs which are used to estimate the control state of present sampling period under the vacant sampling cases. The comprehensive control methods simplify control design which is easier to be implemented in engineering. The performance of the systems is also improved. Simulation results verify the validity of the proposed theory.

## 1. Introduction

Feedback control systems in which control loops are closed through a real-time network are called networked control systems (NCSs) [1, 2]. This type of systems has many advantages such as flexible system design, simple installation and maintenance, increased system agility, and reduced system wiring. Compared with conventional point-to-point control systems, however, the insertion of communication network in the feedback control loop makes the analysis and design of NCSs more complex. Conventional control theory with many ideal assumptions, such as synchronized control and non-delayed sensing and actuation, has to be reevaluated before it is applied to NCSs.

The existence of network in NCSs inevitably induces some nondeterministic phenomena, especially network-induced delay, which will degrade the performance of control systems and even destabilize the systems. The network-induced delay mainly comes from two resources: sensor-to-controller delay and controller-to-actuator delay. It is of great significance to explore suitable control methods under the stochastic delay in NCSs to improve the system performance.

Some researchers studied stochastic time-delay and the optimal controllers of NCSs whose network-induced delay was shorter than a sampling period [3–5]. Nevertheless, the network-induced delay is longer than one sampling period in many cases.

Substantial efforts have been done for the nonlinear NCSs with time-varying delay. A time-based neuron-dynamic-programming (NDP) optimal control scheme for uncertain nonlinear NCSs was introduced by using output feedback and without utilizing value and policy iterations [6]. Closed-loop stability in the mean was demonstrated by selecting novel neural-network (NN) update laws. However, the stochastic characteristic of time-delay was not referred in the optimal control design. Cao [7] provided improved time-delay-dependent stability criteria for multi-input and multi-output (MIMO) NCSs with nonlinear perturbations. Related control methods and simulations were not given to support the proposed theory. A high frequency NCS was described by a time-varying delayed delta operator system with a high frequency constraint [8]. An improved stability condition was given for the delta operator system by using a generalized Kalman-Yakubovic-Popov lemma. They did not discuss the stochastic characteristic of time-delay and concrete control methods. In [9], optimizing controller design of real-time NCSs was presented based on different models. For time-delay less than one sampling interval, they modeled the system as a time-invariant control system with constant time-delay. For time-delay greater than one sampling interval, they modeled it as a jump linear control system. Nevertheless, NCSs models should be nonlinear because of time-varying delay. Using the Lyapunov-Krasovskii method, a sufficient condition for asymptotic stability of nonlinear NCSs was provided in [10]. Measured values were asynchronously sampled and transmitted over multiple communications links. The effects of communication in each link were captured by a time-varying delay element. In order to avoid complexities of these kinds of nonlinear NCSs, an assumption of delay bound was made under which the NCSs models were not sensitive to asynchrony of sampling and transmission between different links.

With regard to the characteristic of uncertain delay in NCSs, some researchers proposed predictive control methods to compensate time-delay whose probability distribution was unknown [11–14]. The stochastic time-delay system was transformed to a deterministic delay system by placing a special amount of buffers at the nodes in NCSs. Multistep predicting controllers were designed to improve system performance through model matching and multistep predictive output compensation. However, this kind of models is too complex and has high computational cost due to the uncertainty of time-varying delay.

When network-induced delay has a known probability distribution and is longer than one sampling period, Yu et al. [15] proposed a control mode: sensor and actuator were time-driven and controller was event-driven. Ma and Fang [16] put forward a time-division control mode: sensor was time-driven, controller was event-driven, and actuator was time-division-driven. Under the two kinds of control modes, perhaps there were not new control inputs arriving at the actuator during a sampling period because of data congestion or network bandwidth limitation. In this case the old control input of last sampling period continued acting on the plant, which might induce long time-delay. This kind of situation is called vacant sampling. The vacant sampling in NCSs may degrade the performance of control systems and even destabilize the systems. Furthermore, it will make optimal controller design difficult to be implemented in engineering when the time-delay of NCSs is too long. It is important to take some measures for compensating vacant sampling and long time-delay.

To solve all the aforementioned problems, comprehensive control methods of NCSs are proposed in this paper. Time-delay compensation control and linear-quadratic-Guassian (LQG) optimal control are adopted and the systems switch different controllers between two different states. LQG optimal controller is used with probability 1 − *α* in normal state, which is shown to render the systems mean square exponentially stable and guarantees the performance of NCSs with big probability. Combined with the strength of predictive control methods, time-delay compensation controller is used with probability *α* in abnormal state with long delay and compensates vacant sampling and long time-delay. The stochastic characteristic of time-delay is also considered to design the optimal control of NCSs. The proposed comprehensive control methods reduce the complexities of system control and computational cost under the guarantee of improving system performance.

The rest of this paper is organized as follows.  Section 2 describes comprehensive control methods of NCSs. Simulations are given in Section 3, followed by conclusions in Section 4.

## 2. Description of Comprehensive Control Methods

The stochastic delay in NCSs mainly comes from two resources: sensor-to-controller delay *τ*_sc_ and controller-to-actuator delay *τ*_ca_. Assume that network-induced delay *τ* has a known probability distribution function and *τ* = *τ*_sc_ + *τ*_ca_ ≤ *pT* (*p* is a positive integer, *p* ≥ 2, and *T* is the sampling period of sensor). Now, we describe the model of NCSs. We assume the state equation of plant is linear time-invariant which is expressed as

(1) $$\begin{array}{c} \overset{\cdot }{x(t)}=Ax\left(t\right)+Bu\left(t\right),\;\;y\left(t\right)=Cx\left(t\right), \end{array}$$

where *x*(*t*) ∈ *R* ^*n*^,   *u*(*t*) ∈ *R* ^*m*^. *A*, *B*, and *C* are matrices of appropriate sizes. Discretizing (1) separately under Yu's control mode [15] and Ma's control mode [16] over a sampling interval [*kT*, (*k* + 1)*T*), we have a stochastic NCSs model as follows:

(2) $$\begin{array}{c} x_{k+1}=A_{c}x_{k}+\overset{p}{\underset{j=0}{\sum }}B_{j}^{k}u_{k-j},\;\;y_{k}=Cx_{k}, \end{array}$$

where under Yu's control mode:

(3) $$\begin{array}{c} x_{k}=x\left(kT\right),\;\;y_{k}=y\left(kT\right),\;\;A_{c}=e^{AT},\\ B_{j}^{k}=\beta_{j}\Gamma =\beta_{j}\overset{T}{\underset{0}{\int }}e^{At}dt\cdot B,\\ \beta_{0},\ldots ,\beta_{p}\in \left\{0,1\right\}\;\left(j=0,\ldots ,p\right),\;\;\; \end{array}$$

and under Ma's control mode:

(4) xk=x(kT),  yk=y(kT),  Ac=eAT,Bjk=∫0T/(p+1)eA(T−s)×(αpje−Atp+1k+⋯+αije−Ati+1k+⋯+α0je−At1k)ds·B, αi0,…,αip∈{0,1},∑j=0pαij=1, (i=0,…,p,j=0,…,p),

*B*_*j*_^*k*^  (*j* = 0,…, *p*) are stochastic variables.

Assume that the transfer delay from sensor to actuator at the moment *kT* is *d*_*k*_  (0 ⩽ *d*_*k*_ ⩽ *p*). We can conclude that {*d*_*k*_, *k* = 0,1, 2,…} is a Markov chain. The state transition matrixes under Yu's control mode and Ma's control mode can be derived [15, 16].

Under the two kinds of control modes above, perhaps there is vacant sampling during a sampling period because of data congestion or network bandwidth limitation. For the sake of avoiding the vacant sampling in NCSs, time-delay compensation control is put forward and used to compensate long time-delay in this paper. It is described as follows.

A buffer window is established at actuator to store some history control inputs {*u*_*k*−*p*_, *u*_*k*−*p*+1_,…, *u*_*k*−1_}. There are at most *p* history control inputs in the window. If vacant sampling occurs in NCSs over a sampling interval [*kT*, (*k* + 1)*T*), the history control inputs are used to estimate the control state of present sampling period. According to the time order of history control inputs, some weights are derived. The average value of history control inputs with weights is calculated to act on plant instead of the old control input of last sampling period. In terms of the synthetic effects of these history control inputs, the performance of NCSs is improved. At the beginning, the initial values of control inputs in the buffer window are set as 0. We use the following equation to estimate control input which is denoted as *u*_*k*_ over a sampling interval [*kT*, (*k* + 1)*T*). The estimation value of *u*_*k*_ is denoted by

(5) $$\begin{array}{c} {\hat{u}}_{k}=\{\begin{array}{cc} \frac{\sum_{i=1}^{k-1}a_{i}u_{i}}{k-1}, & \left(k<p\right),\\ \frac{\sum_{j=k-p}^{k-1}b_{j}u_{j}}{p}, & \left(k\geq p\right), \end{array} \end{array}$$

where {*a*_*i*_, *i* = 1,…, *k* − 1},  {*b*_*j*_, *j* = *k* − *p*,…, *k* − 1} are weight sequences for history control inputs. They are calculated as follows:

(6) $$\begin{array}{c} a_{i}=\{\begin{array}{cc} 0.5, & \left(i=1,\ldots ,\left[\frac{k-1}{2}\right]\right),\\ 1.5, & \left(i=\left[\frac{k-1}{2}\right]+1,\ldots ,k-1\right), \end{array} \end{array}$$

(7) $$\begin{array}{c} b_{j}=\{\begin{array}{cc} 0.5, & \left(j=k-p,\ldots ,k-\left[\frac{p}{2}\right]\right),\\ 1.5, & \left(j=k-\left[\frac{p}{2}\right]+1,\ldots ,k-1\right), \end{array} \end{array}$$

where [(*k* − 1)/2] is the integer function of (*k* − 1)/2 and [*p*/2] is the integer function of *p*/2. The time-delay compensation control guarantees not only the priority of history control inputs with smaller delay but also the synthetic effects of all history control inputs.

In NCSs, optimal control is often used to improve system performance and stabilize the whole system. Now we design the LQG optimal controller of system (2).

**Theorem 1 .**

With the system having full state information, the LQG optimal control law for NCSs system (2) is

(8) $$\begin{array}{c} u_{k}=-L_{k}{\left[x_{k}^{T},u_{k-p}^{T},u_{k-p+1}^{T},\ldots ,u_{k-1}^{T}\right]}^{T}, \end{array}$$

where

(9) $$\begin{array}{c} L_{k}={\left[E\left(B_{k}^{T}S_{k+1}B_{k}\right)+R^{\prime }\right]}^{-1}\cdot E\left(B_{k}^{T}S_{k+1}A_{k}\right),\\ S_{k}=E\left(A_{k}^{T}S_{k+1}A_{k}\right)+Q^{\prime }-L_{k}^{T}\left[E\left(B_{k}^{T}S_{k+1}B_{k}\right)+R^{\prime }\right]L_{k}, \end{array}$$

and it renders system (2) mean square exponentially stable.

**Proof**

In this paper, we design a control law of the stochastic open-loop NCSs as expressed in (2) to minimize the cost function

(10) $$\begin{array}{c} J_{N}=E\left\{x_{N}^{T}P_{N}x_{N}+\overset{N-1}{\underset{k=0}{\sum }}\left[x_{k}^{T}Qx_{k}+u_{k}^{T}Ru_{k}\right]\right\}, \end{array}$$

where *P*_*N*_ and *Q* are symmetric and positive semidefinite and *R* is symmetric and positive definite. At first we introduce a new state variable

(11) $$\begin{array}{c} z_{k}={\left[x_{k}^{T},u_{k-p}^{T},u_{k-p+1}^{T},\ldots ,u_{k-1}^{T}\right]}^{T}\in R^{n+pm}. \end{array}$$

Then system (2) can be expressed as follows:

(12) $$\begin{array}{c} z_{k+1}=A_{k}z_{k}+B_{k}u_{k}, \end{array}$$

where

(13) $$\begin{array}{c} A_{k}=\left[\begin{array}{cccccc} A_{c} & B_{p}^{k} & B_{p-1}^{k} & \cdots & B_{2}^{k} & B_{1}^{k}\\ 0 & 0 & I_{m} & \cdots & 0 & 0\\ \vdots & \vdots & \vdots & \ddots & 0 & 0\\ 0 & 0 & 0 & \cdots & 0 & I_{m}\\ 0 & 0 & 0 & \cdots & 0 & 0 \end{array}\right],\\ B_{k}=\left[\begin{array}{c} B_{0}^{k}\\ 0\\ \vdots \\ 0\\ I_{m} \end{array}\right]. \end{array}$$

Cost function (10) is equivalent to

(14) $$\begin{array}{c} J_{N}=E\left\{z_{N}^{T}P_{N}^{\prime }z_{N}+\overset{N-1}{\underset{k=0}{\sum }}\left[z_{k}^{T}Q^{\prime }z_{k}+u_{k}^{T}R^{\prime }u_{k}\right]\right\}, \end{array}$$

where

(15) $$\begin{array}{c} P_{N}^{\prime }=\left[\begin{array}{ccccc} P_{N} & 0 & 0 & \cdots & 0\\ 0 & \left(\frac{1}{\left(p+1\right)}\right)R & 0 & \cdots & 0\\ 0 & 0 & \left(\frac{2}{\left(p+1\right)}\right)R & \cdots & 0\\ \vdots & \vdots & \vdots & \ddots & 0\\ 0 & 0 & 0 & \cdots & \left(\frac{p}{\left(p+1\right)}\right)R \end{array}\right],\\ Q^{\prime }=\left[\begin{array}{ccccc} Q & 0 & 0 & \cdots & 0\\ 0 & \left(\frac{1}{\left(p+1\right)}\right)R & 0 & \cdots & 0\\ 0 & 0 & \left(\frac{1}{\left(p+1\right)}\right)R & \cdots & 0\\ \vdots & \vdots & \vdots & \ddots & 0\\ 0 & 0 & 0 & \cdots & \left(\frac{1}{\left(p+1\right)}\right)R \end{array}\right],\\ R^{\prime }=\left(\frac{1}{\left(p+1\right)}\right)R. \end{array}$$

Now *P*_*N*_′ and *Q*′ are symmetric and positive semidefinite and *R*′ is symmetric and positive definite.

Minimizing cost function (14) is equivalent to minimizing cost function (10). At first we write out Bellman functional equation of cost function (14). Consider

(16) min⁡⁡Jk=min⁡⁡uk,…,uN−1E{zNTPN′zN+∑l=kN−1[zlTQ′zl+ulTR′ul]}=E{min⁡⁡uk,…,uN−1{zNTPN′zN+∑l=kN−1[zlTQ′zl+ulTR′ul]}}=E{min⁡⁡uk,…,uN−1E{zNTPN′zN +∑l=kN−1[zlTQ′zl+ulTR′ul] ∣ zk}=E[V(zk,k)],V(zk,k)=min⁡uk⁡E{zkTQ′zk+ukTR′uk+min⁡uk+1…,uN−1⁡E{zNTPN′zN+∑l=k+1N−1[zlTQ′zl+ulTR′ul] ∣ zk+1} ∣ zk}=min⁡⁡uk E{zkTQ′zk+ukTR′uk+V(zk+1,k+1) ∣ zk}=min⁡⁡uk{zkTQ′zk+ukTR′uk+E{V(zk+1,k+1) ∣ zk}}.  

Equation (16) is Bellman functional equation.

Then, we prove that the solution of (16) is as follows:

(17) $$\begin{array}{c} V\left(z_{k},k\right)=z_{k}^{T}S_{k}z_{k}+s_{k}, \end{array}$$

where *S*_*k*_ and *s*_*k*_ are nondeterministic.

We prove (17) with mathematical induction. Let *q* express time. When *q* = *N*, the conclusion is apparently correct. If we suppose that when *q* = *k* + 1 the conclusion is correct, we will prove that when *q* = *k* the conclusion is also correct. Consider

(a) *q* = *N*
  (18) $$\begin{array}{c} V\left(z_{N},N\right)=\underset{u_{N}}{\min}\;E\left\{z_{N}^{T}P_{N}^{\prime }z_{N}\;|\;z_{N}\right\}=z_{N}^{T}P_{N}^{\prime }z_{N}. \end{array}$$
 Let *S*_*N*_ = *P*_*N*_′ and *s*_*N*_ = 0 and then (17) holds.
(b) When *q* = *k* + 1, (17) holds. Now, we have
  (19) $$\begin{array}{c} V\left(z_{k+1},k+1\right)=z_{k+1}^{T}S_{k+1}z_{k+1}+s_{k+1},\\ E\left\{V\left(z_{k+1},k+1\right)\;|\;z_{k}\right\}=E\left\{z_{k+1}^{T}S_{k+1}z_{k+1}\;|\;z_{k}\right\}+s_{k+1}. \end{array}$$
 Using (12) we can conclude
  (20) E{V(zk+1,k+1) ∣ zk} =E{(Akzk+Bkuk)T·Sk+1   ·(Akzk+Bkuk) ∣ zk}+sk+1 =(Akzk+Bkuk)T·Sk+1·(Akzk+Bkuk)     +tr⁡ Sk+1R1+sk+1,
 where *R*_1_ = *E*{(*z*_*k*+1_ − *E*{*z*_*k*+1_})(*z*_*k*+1_−*E*{*z*_*k*+1_})^*T*^}.
(c) *q* = *k*
 Using (20) and considering (16), we can derive
  (21) V(zk,k) =min⁡⁡uk E{zkTQ′zk+ukTR′uk+E{(Akzk+Bkuk)T·Sk+1·(Akzk+Bkuk) ∣ zk}+sk+1} =min⁡⁡uk{zkTSkzk+[uk+Lkzk]T·[E(BkTSk+1Bk)+R′]·[uk+Lkzk]+tr⁡Sk+1R1+sk+1},
 where
  (22) $$\begin{array}{c} L_{k}={\left[E\left(B_{k}^{T}S_{k+1}B_{k}\right)+R^{\prime }\right]}^{-1}\cdot E\left(B_{k}^{T}S_{k+1}A_{k}\right),\\ S_{k}=E\left(A_{k}^{T}S_{k+1}A_{k}\right)+Q^{\prime }-L_{k}^{T}\left[E\left(B_{k}^{T}S_{k+1}B_{k}\right)+R^{\prime }\right]L_{k},\\ s_{k}=\mathrm{tr}S_{k+1}R_{1}+s_{k+1}. \end{array}$$
 Letting *u*_*k*_ = −*L*_*k*_ *z*_*k*_, *V*(*z*_*k*_, *k*) is the minimum cost. Consider
  (23) $$\begin{array}{c} V\left(z_{k},k\right)=z_{k}^{T}S_{k}z_{k}+s_{k}. \end{array}$$
 Thus, when *q* = *k*, (17) also holds, and when
  (24) uk=−Lkzk=−Lk[xkT,uk−pT,uk−p+1T,…,uk−1T]T,

*V*(*z*_*k*_, *k*) is minimum cost, so is *J*_*k*_.

Similar to the proving process in [17], we can conclude that LQG optimal control law (8) renders system (2) mean square exponentially stable. Now we prove Theorem 1.

In practical application of NCSs, network-induced delay is often longer than one sampling period. When the time-delay of NCSs is too long, it will make optimal controller design difficult to be implemented in engineering. In order to reduce the complexities and computational cost of system control, comprehensive control methods are proposed whose main idea is as follows.

When time-delay is smaller than a suitable delay bound, optimal controller (8) is used to stabilize the systems during most of running time, which can make optimal control easier to be implemented. While time-delay is bigger than the delay bound, time-delay compensation controller (5) is used to compensate vacant sampling and long time-delay.

The comprehensive control methods are based on *α* confidence level in this paper. Normal and abnormal states are defined for the delay's two different cases. The related definitions are given as follows.

**Definition 2 (see [18]).**

Assume that the distribution function of population *X* is *F*(*x*; *θ*), *θ* is an unknown parameter, and *θ* ∈ Θ. For a constant *α*  (0 < *α* < 1), if statistic variable $\bar{\theta }=\bar{\theta }(X_{1},X_{2},\ldots ,X_{n})$, which is derived from the samples *X*_1_, *X*_2_,…, *X*_*n*_, meets the following equation:

(25) $$\begin{array}{c} P\left\{\theta <\bar{\theta }\left(X_{1},X_{2},\ldots ,X_{n}\right)\right\}=1-\alpha , \end{array}$$

then stochastic interval $\left(-\infty \;\bar{\theta }\right)$ is called single-side confidence interval with confidence level 1 − *α*. $\bar{\theta }$ that is called single-side confidence upper limit.

Now we discuss the single-side confidence interval of time-delay *τ* based on confidence level *α* in NCSs. For given constant *α*  (0 < *α* < 1), the single-side confidence upper limit $\bar{\tau }=p^{\prime }T$  (*p*′ is a positive integer and *p*′ < *p*) of *τ* can be derived. On the basis of *τ* and $\bar{\tau }$, normal and abnormal states are defined as follows.

**Definition 3 .**

When $\tau <\bar{\tau }$ and $P\left\{\tau <\bar{\tau }\right\}=1-\alpha$, the system state is called normal state. When $\bar{\tau }\leq \tau \leq pT$ and $P\left\{\bar{\tau }\leq \tau \leq pT\right\}=\alpha$, the system state is called abnormal state.

In NCSs, time-delay *τ* does not always access or reach the maximum delay *pT*. In general cases, it is near expectation value *E*{*τ*} with big probability. We can choose suitable confidence level *α* to make random event $\left\{\tau <\bar{\tau }\right\}$ be a big-probability event when random event $\left\{\bar{\tau }\leq \tau \leq pT\right\}$ is a small-probability event. The probability of the systems in normal state is 1 − *α*. LQG optimal controller (8) is adopted in normal state, which is shown to render the systems mean square exponentially stable during most of running time. The probability of the systems in abnormal state is *α*. Time-delay compensation controller (5) is adopted in abnormal state to compensate vacant sampling and long time-delay.

Using Schur complement and Cone-complement linear technique, an approximate solution of control law was obtained in [19], which rendered system (2) asymptotically stable. It is described as follows.

**Theorem 4 .**

If there exist  *P* > 0,  *M* > 0,  *Q* > 0,  and *N* > 0, *G*,  *X*,  *Y*,  *Ζ*, such that

(26) $$\begin{array}{c} \left[\begin{array}{cccc} l_{11} & -Y & A_{k}^{T} & l_{41}^{T}\\ -Y^{T} & -Q & B_{k}^{T} & l_{42}^{T}\\ A_{k} & B_{k} & -M & 0\\ l_{41} & l_{42} & 0 & -\left(p-1\right)N \end{array}\right]<0,\\ \left[\begin{array}{cc} X & Y\\ Y^{T} & Ζ \end{array}\right]\geq 0,\\ \left[\begin{array}{cc} P & I_{pn}\\ I_{pn} & M \end{array}\right]\geq 0,\\ \left[\begin{array}{cc} Ζ & I_{pn}\\ I_{pn} & N \end{array}\right]\geq 0, \end{array}$$

where

(27) $$\begin{array}{c} l_{11}=-P+\left(p-1\right)X+Y+Y^{T}+Q,\\ l_{41}=\left(p-1\right)\left(A_{k}-I_{pn}\right),\\ l_{42}=\left(p-1\right)B_{k}, \end{array}$$

then system (2) is asymptotically stable for any network-induced delay *τ* satisfying 0 ≤ *τ* ≤ *pT* (*p* ≥ 2 is a positive integer).

**Proof**

See proof of Theorem  1 in [19].

When the maximum delay *pT* is known, the controller

(28) $$\begin{array}{c} u_{k}=Gx_{k} \end{array}$$

can be obtained based on Theorem 4 using the MATLAB LMI Toolbox.

Now we make some changes in the comprehensive control methods. LQG optimal controller (8) is adopted in normal state, which is shown to render the systems mean square exponentially stable. Controller (28) is adopted in abnormal state to compensate vacant sampling and long time-delay, which is shown to approximately render the systems asymptotically stable. Then the system model is described as follows.

We denote the state variable and output of NCSs in normal state and abnormal state as *x*_*k*_^0^,  *y*_*k*_^0^,  *x*_*k*_^*f*^, and  *y*_*k*_^*f*^. Then the optimal controller in Theorem 1 can be written as

(29) uk=−Lkzk=−[Lk0Lkp′Lkp′−1⋯Lk1] ·[xk0uk−p′uk−p′+1⋮uk−1],

where *L*_*k*_^0^ ∈ *R* ^1×*n*^ and  *L*_*k*_^*i*^ ∈ *R* ^1×*m*^  (*i* = 1,…, *p*′). Time-delay compensating controller (28) can be written as

(30) $$\begin{array}{c} u_{k}=Gx_{k}^{f}. \end{array}$$

From the discussion above, we get the comprehensive control model of system (2)

(31) x¯k+1=A¯0x¯k+∑j=1p′A¯jx¯k−j +∑j=p′+1pB¯jx¯k−j+∑j=12p′D¯juk−j,y¯k+1=C¯x¯k+1,         

where

(32) $$\begin{array}{c} {\bar{x}}_{k}=\left[\begin{array}{c} x_{k}^{0}\\ x_{k}^{f} \end{array}\right],\;\;{\bar{A}}_{0}=\left[\begin{array}{cc} A_{c}-L_{k}^{0}B_{0}^{k} & 0\\ 0 & A_{c}+GB_{0}^{k} \end{array}\right],\\ {\bar{A}}_{j}=\left[\begin{array}{cc} -L_{k}^{0}B_{j}^{k} & 0\\ 0 & GB_{j}^{k} \end{array}\right],\;\;\bar{C}=\left[\begin{array}{cc} C & 0\\ 0 & C \end{array}\right],\\ {\bar{B}}_{j}=\left[\begin{array}{cc} 0 & 0\\ 0 & I_{n} \end{array}\right],\;\;{\bar{D}}_{j}=\left[\begin{array}{cc} D_{j}^{k} & 0\\ 0 & 0 \end{array}\right],\\ D_{j}^{k}=\{\begin{array}{cc} -\overset{j-1}{\underset{i=0}{\sum }}B_{i}^{k}L_{k}^{j-i}, & \left(1\leq j\leq p^{\prime }\right)\text{,}\\ -\overset{p^{\prime }}{\underset{i=j-p^{\prime }}{\sum }}B_{i}^{k}L_{k}^{j-i}, & \left(p^{\prime }+1\leq j\leq 2p^{\prime }\right). \end{array} \end{array}$$

## 3. Simulations

The simplified model of the inverted pendulum process is as follows [20]:

(33) x(t)·=[0110]x(t)+[01]u(t),y(t)=[10]x(t).

In this paper, we use MatLab and C++ to simulate the comprehensive control methods on the networked inverted pendulum system that we construct based on NS2 [21].

In the simulations, parameters are selected as follows: *T* = 0.05 s, $P_{N}=Q=\left[\begin{array}{cc} 1 & 0\\ 0 & 1 \end{array}\right]$, and *R* = 0.1. It is assumed that the maximum network-induced delay is 3*T*; that is, *p* = 3. Using stochastic sampling experiment, we can get $\bar{\tau }=2T$ (*p*′ = 2) under confidence level parameter *α* = 0.05. The state transition matrix under Yu's control mode is

(34) $$\begin{array}{c} Q_{M}=\left[\begin{array}{ccc} 0.8 & 0.2 & 0\\ 0.4 & 0.1 & 0.5\\ 0.4 & 0.1 & 0.5 \end{array}\right]. \end{array}$$

The state transition matrixes under Ma's control mode are

(35) $$\begin{array}{c} P_{M_{1}}=\left[\begin{array}{ccc} 1 & 0 & 0\\ 0.88 & 0.12 & 0\\ 0.82 & 0.08 & 0.1 \end{array}\right],\\ P_{M_{2}}=\left[\begin{array}{ccc} 1 & 0 & 0\\ 0.8889 & 0.1111 & 0\\ 0.8 & 0.1 & 0.1 \end{array}\right]. \end{array}$$

Then by Theorem 1 we can get the optimal control input

(36) $$\begin{array}{c} u_{k}=-\left[\begin{array}{cc} 3.9760 & 3.9679 \end{array}\right]x_{k}-0.0085u_{k-2}-0.0206u_{k-1}. \end{array}$$

**Figure 1:**
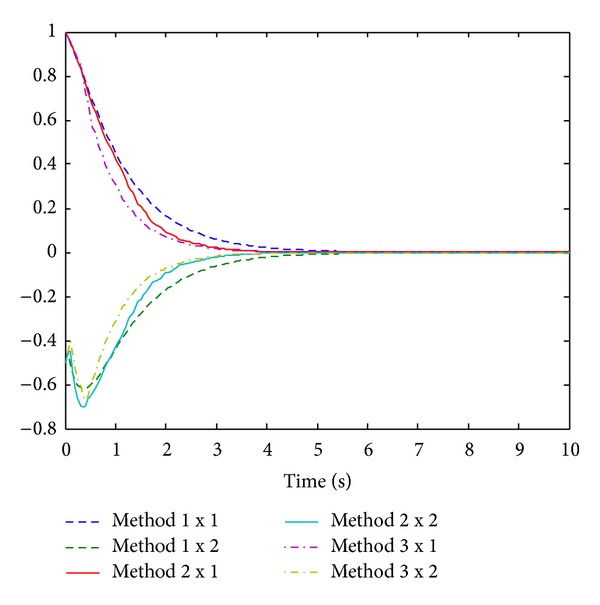
Curves of state response.

At first we use controller (5) as time-delay compensation controller. With the initial state value *x*(0) = [1 −0.5]^*T*^ of the system, the simulation results of method 1 (method without considering comprehensive control methods under Ma's control mode), method 2 (comprehensive control methods under Yu's control mode), and method 3 (comprehensive control methods under Ma's control mode) are given in Figure 1.

**Figure 2:**
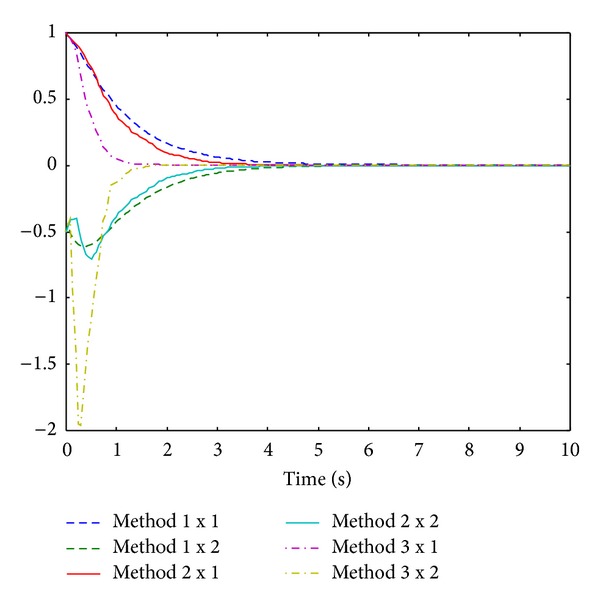
Curves of state response.

Then we use controller (28) as time-delay compensation controller. By Theorem 4, we can obtain the control input *u*_*k*_ = −[4.0571 4.0553]*x*_*k*_. With the initial state value *x*(0) = [1 −0.5]^*T*^ of the system, the simulation results of method 1 (method without considering comprehensive control methods under Yu's control mode), method 2 (comprehensive control methods under Yu's control mode), and method 3 (comprehensive control methods under Ma's control mode) are given in Figure 2.

From Figures 1 and 2, we can see that the system performance is obviously improved under the comprehensive control methods which make NCSs faster to reach stability status. The simulation results show the validity of the proposed theory.

## 4. Conclusions

In order to improve the performance of NCSs with multistep delay, comprehensive control methods based on confidence level are presented in this paper.

Time-delay compensation control and LQG optimal control are adopted and the systems switch different controllers between two different states. LQG optimal control is used with probability 1 − *α* in normal state, which is shown to render the systems mean square exponentially stable. Time-delay compensation control is used with probability *α* in abnormal state. The comprehensive control methods simplify controller design and reduce computational cost. It is proved by simulations that the new control methods have better control effects than single control method. We will synthesize more optimal control methods in future work to further improve the performance of NCSs.
